# The application value of support vector machine model based on multimodal MRI in predicting IDH-1mutation and Ki-67 expression in glioma

**DOI:** 10.1186/s12880-024-01414-1

**Published:** 2024-09-16

**Authors:** He-Xin Liang, Zong-Ying Wang, Yao Li, An-Ning Ren, Zhi-Feng Chen, Xi-Zhen Wang, Xi-Ming Wang, Zhen-Guo Yuan

**Affiliations:** 1 Department of Radiology, Shandong Provincial Hospital Affiliated to Shandong First Medical University, Jinan, China; 2 Shandong Provincial Hospital Affiliated to Cheeloo College of Medicine of Shandong University, Jinan, China; 3 Cancer Hospital, Affiliated to Shandong First Medical University, Jinan, China; 4 Department of Radiology, Weifang People’s Hospital, Weifang, China; 5 Zhucheng People’s Hospital, Weifang, China; 6 Medical Imaging Center of the Affiliated Hospital of Weifang Medical University, Weifang, China; 7 Monash Biomedical Imaging, Monash University, Clayton, VIC Australia; 8 Department of Data Science & AI, Faculty of IT, Monash University, Clayton, VIC Australia

**Keywords:** Glioma, IDH-1, Ki-67, Support vector machine

## Abstract

**Purpose:**

To investigate the application value of support vector machine (SVM) model based on diffusion-weighted imaging (DWI), dynamic contrast-enhanced (DCE) and amide proton transfer- weighted (APTW) imaging in predicting isocitrate dehydrogenase 1(IDH-1) mutation and Ki-67 expression in glioma.

**Methods:**

The DWI, DCE and APTW images of 309 patients with glioma confirmed by pathology were retrospectively analyzed and divided into the IDH-1 group (IDH-1(+) group and IDH-1(-) group) and Ki-67 group (low expression group (Ki-67 ≤ 10%) and high expression group (Ki-67 > 10%)). All cases were divided into the training set, and validation set according to the ratio of 7:3. The training set was used to select features and establish machine learning models. The SVM model was established with the data after feature selection. Four single sequence models and one combined model were established in IDH-1 group and Ki-67 group. The receiver operator characteristic (ROC) curve was used to evaluate the diagnostic performance of the model. Validation set data was used for further validation.

**Results:**

Both in the IDH-1 group and Ki-67 group, the combined model had better predictive efficiency than single sequence model, although the single sequence model had a better predictive efficiency. In the Ki-67 group, the combined model was built from six selected radiomics features, and the AUC were 0.965 and 0.931 in the training and validation sets, respectively. In the IDH-1 group, the combined model was built from four selected radiomics features, and the AUC were 0.997 and 0.967 in the training and validation sets, respectively.

**Conclusion:**

The radiomics model established by DWI, DCE and APTW images could be used to detect IDH-1 mutation and Ki-67 expression in glioma patients before surgery. The prediction performance of the radiomics model based on the combination sequence was better than that of the single sequence model.

## Introduction

Gliomas are the most common malignancies of the nervous system [1]. In the 2021 World Health Organization (WHO) brain tumor classification of central nervous system, isocitrate dehydrogenase (IDH) plays an important role in the classification of glioma [2]. Compared with IDH-1 wild-type (IDH-1(-)), the glioma patients with IDH-1 mutant-type (IDH-1(+)) have longer survival time and better efficacy of chemoradiotherapy [3]. Not only IDH-1, Ki-67 index is also an important biomarker of the tumor’s biological behavior. The expression level of Ki-67 is closely related to the survival rate of patients. The higher expression of Ki-67, the lower prognosis of patients [4]. Therefore, preoperative evaluation of Ki-67 expression and IDH-1 mutation in glioma is of great significance for formulating treatment plans and evaluating prognosis.

MRI is a common technique for noninvasive diagnosis of glioma before surgery, but conventional MRI mainly provides anatomical information about the tumor. Functional magnetic resonance imaging (fMRI) plays an important role in evaluating tumor molecular metabolism. Dynamic contrast-enhanced (DCE) MRI is a common MR perfusion imaging technique, which can quantitatively evaluate the density and permeability of microvessels, and the proliferation of tumor cells in glioma using volume transfer constant (Ktrans) and extravascular extracellular volume fraction (Ve). A previous study had demonstrated the clinical potential of DCE-MRI in the evolution of glioma IDH-1 mutation [5]. Diffusion-weighted imaging (DWI) can assess the cell density and provide complementary biophysical information about tumor microstructure [6]. Aminoacyl proton transfer-weighted (APTW) is a contrast agent-free MRI method and can assess the amount of mobile proteins or peptides in the tumor tissue [7]. APTW imaging has become a promising method for predicting the glioma grade and IDH mutation status [8]. Radiomics is to mine high-throughput quantitative image features from standard medical imaging, extract data and apply them to clinical practice. It can improve the accuracy of diagnosis and prognosis prediction and is becoming increasingly important in cancer research [9]. Radiomics has been widely used to develop differential and prognostic models, especially in brain tumors, such as classification and genotyping of gliomas, differentiation of gliomas from metastases, prediction of prognosis of gliomas, etc. [10–12].

But even when combined with radiomics, conventional MRI provides limited information for evaluating Ki-67 expression and IDH-1 mutation. To this end, we aimed to develop and validate radiomics models based on DWI, DCE, and APTW sequences to evaluate Ki-67 expression and IDH-1 mutation in gliomas.

## Materials and methods

### Subjects

The study was approved by the hospital’s institutional review board, and informed consent was waived, given the retrospective study and anonymised data of patients. We retrospectively collected all patients’ clinical information and MRI data from January 2018 to March 2022. According to the 2021 WHO brain tumor classification of central nervous system, patients with postoperative pathological diagnosis of glioma were included. The inclusion criteria were: (1) availability of pathological analysis reports; (2) complete preoperative MRI data, including CE-T1WI, DWI, DCE, and APTW sequences; (3) over 18 years old. A total of 357 patients were enrolled in the study. Then, 48 patients were excluded because of (1) lack of IDH-1 gene or Ki-67 expression (*n* = 31), (2) motion artifacts on MRI or other artifacts that could influence the quality of image severely (*n* = 6), (3) postoperative patients, and previous radiation or chemotherapy (*n* = 11). Finally, 309 eligible subjects were enrolled (123 patients IDH-1(+), 186 patients IDH-1(-), 132 patients with low expression of Ki-67, and 177 patients with high expression of Ki-67).

The information of IDH-1 and Ki-67 was obtained from the pathology department of the hospital. Standard immunohistochemical staining method was used for postoperative specimens. In the study, according to the percentage of positive staining, Ki-67 was divided into two types: Ki-67 ≤ 10% positive staining was considered as low expression, and Ki-67 > 10% positive staining was regarded as high expression [13]. According to different research purposes, patients were divided into IDH-1 group and Ki-67 group, and 103 patients in each group were stratified and randomly sampled into training set (70%) and validation set (30%) according to the ratio of 7:3.

### MRI experiments

MRI scans were performed using a clinical 3.0T Scanner (Ingenia CX, Philips Healthcare, Best, the Netherlands) with a 32-channel head coil. Conventional MRI including T1WI, T2WI, FLAIR, CE-T1WI were collected. DWI parameters were TR/TE = 2025/71ms, field of view (FOV) = 230 × 230mm^2^, b values = 0, 1000s/mm^2^, matrix = 128 × 98, reconstruction matrix = 256, slice number = 20, slice thickness = 5.5 mm, acquisition time = 12s. Two groups T1-Vibe 3D volume interpolated breath-hold sequence were scanned before contrast injection, with the following parameters: TR/TE = 6.00/2.46ms, flip Angle (FA) = 5°, FOV = 340 × 340mm^2^, matrix = 448 × 448, slice thickness = 1.5 mm. DCE examination was performed with the following scan parameters: TR/TE = 6.00/2.46ms, FA = 10°, FOV = 340 × 340mm^2^, matrix = 448 × 448, slice thickness = 1.5 mm, temporal resolution = 4.5 s, total time = 5 min, total phases = 60. After scanning the first five non-enhanced phases, gadopentate dimeglumine (Gd-DTPA) (Magnevist, Bayer Healthcare, Leverkusen) contrast agent was injected with a high-pressure syringe at a rate of 3mL/s and a total dose of 0.01mmol/kg at the sixth phase, followed by saline irrigation. The clinically approved APTW sequence was performed by using 3D Turbo Spin Echo (TSE) sequences with continuous radio frequency (RF) saturation lasting 2s were used with a TSE factor of 174, TR/TE = 5925/7.8ms, FOV = 230 × 180mm^2^, voxel size = 1.8 × 1.8 × 6mm^3^, matrix = 252 × 220, reconstruction matrix = 256, SENSE acceleration factor = 1.6, slice number = 14, slice thickness = 6 mm, acquisition time = 342s.

### Imaging postprocessing

The DICOM format of ADC maps were acquired by the PACS system. The original DCE images were imported into Siemens Syngo VIA post-processing workstation, and used the MR Tissue 4D module and selected the Tofts model to quantitatively calculate the Ktrans map and Ve map quantitatively. APTW images were post-processed on a Philips workstation (IntelliSpace Portal, Version 7, Philips Healthcare, Best, the Netherlands) to obtain rainbow color-coded images.

### Radiomics analysis

#### Image registration and segmentation

**Fig. 1:**
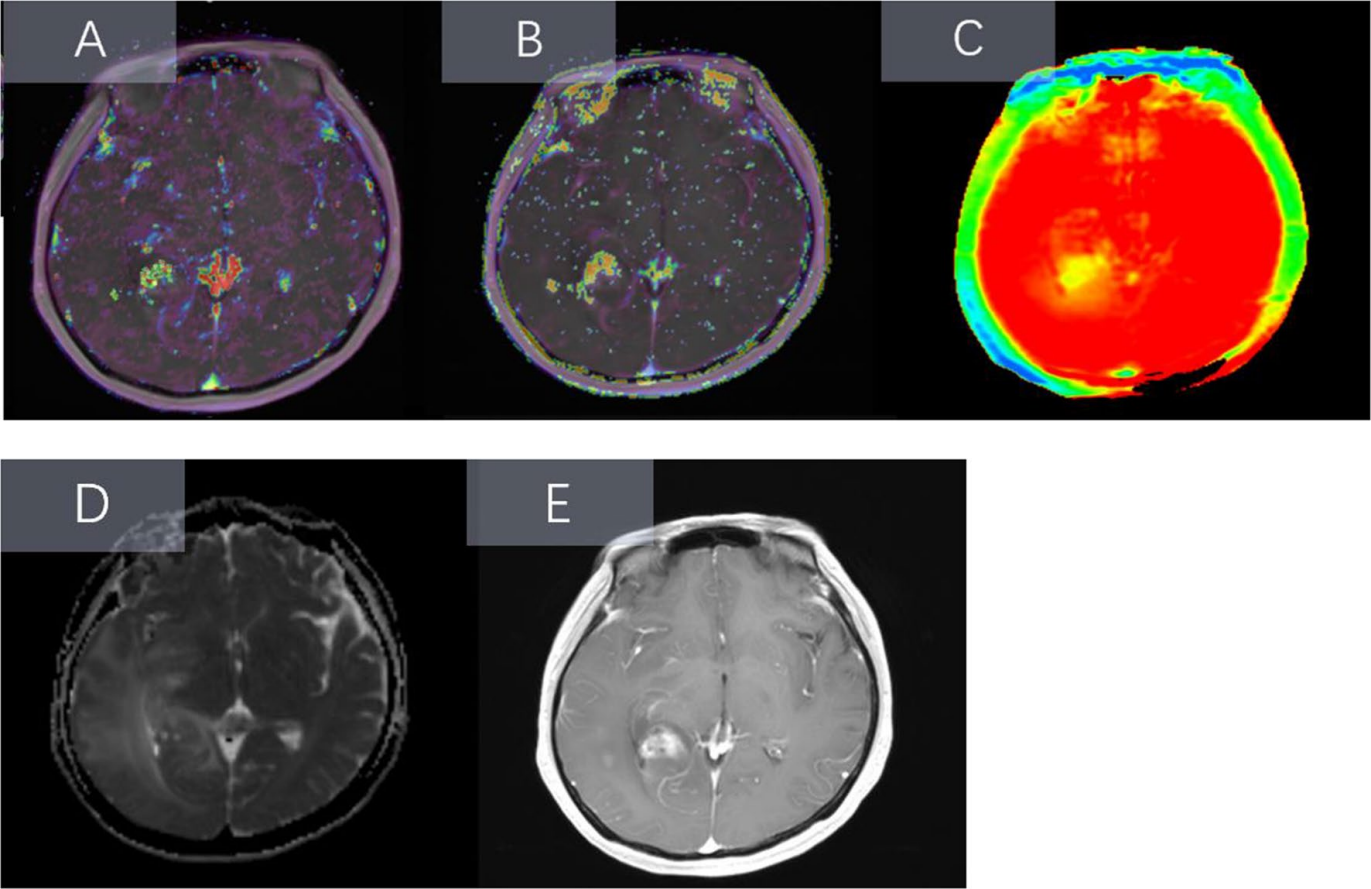
**A** 59-year-old male glioma patient with IDH-1(-) and Ki-67 of 30%. Ktrans (A), Ve (**B**), and APTW (**C**) showed irregular lesions in the left temporal lobe, and the solid part of the tumor was high signal in the corresponding sequence. ADC (**D**) showed that the solid part of the tumor was low signal, and CE-TIWI (E) showed that the solid part of the tumor was significantly enhanced after enhancement

**Fig. 2:**
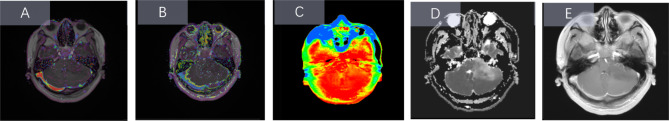
showed a 42-year-old male glioma patient with IDH-1(+) and Ki-67 of 5%. Ktrans (**A**), Ve (**B**), and APTW (**C**) showed irregular lesions in the left cerebellar hemisphere, the solid part of the tumor was low signal in the corresponding sequence. ADC (**D**) showed slightly higher signals intensity in the solid part of the tumor, while CE-T1WI (**E**) showed no significant enhancement in the solid part of the tumor after enhancement

After acquisition, used the 3D-Sliser (version 4.11.2, https://download.slicer.org/) to preprocess the images as follows: (1) Resampling: all images were resampled to 1mm^3^; (2) Imaging registration: ADC, Ktrans, Ve and APTW images were co-registered with CE-T1WI images. And then, sketched the region of interest (ROI). The solid part of the tumor was determined according to the CE-T1WI sequence, and cystic degeneration, necrosis, hemorrhage and peritumoral edema of the tumor were avoided during delineation. Then, the ROI is transferred to the registered ADC maps, Ktrans maps, Ve maps, and APTW maps. The solid components of gliomas are heterogeneous, with different MR signals on different sequences, as shown in Figs. 1 and 2. A radiologist with 10 years of experience performed all segmentations. Radiomics features are extracted from the delineated ROI, so the accuracy of the ROI is critical. To this end, we evaluated the robustness of the features using the intra-group correlation coefficient (ICC). 100 patients were randomly selected and then another radiologist with 10 years of experience delineated the ROI and extracted features, retaining the feature with ICC > 0.8.

#### Features extraction

Features were extracted from ADC, Ktrans, Ve and APTW maps using 3D-Slicer software. 851 features were extracted from each sequence, including (1) 14 shape and size features; (2) 18 first-order statistical features; (3) 75 texture features; (4) 744 wavelet features. A total of 3404 features were extracted from the four sequences. Then we used the Z-Score method to normalize all features.

#### Features selection

To reduce irrelevant and redundant information, Kolmogorov-Smirnov test was first performed on the features of the training set, independent sample t-test was performed on the data conforming to the normal distribution, and Mann-Whitney U test was performed on the data not conforming to the normal distribution. Features with *P* < 0.05 were selected as potential predictive features. Then the least absolute shrinkage and selection operator (LASSO) was used to select features from the training set of single and combined sequences (ADC + Ktrans + Ve + APTW). LASSO could reduce the regression coefficient of features by adjusting the value of penalty coefficient (lambda) to delete unimportant features [14]. In this process, 10 times of cross-validation was carried out to determine the minimum value of lambda.

#### Establishment and evaluation of radiomic models

**Fig. 3:**
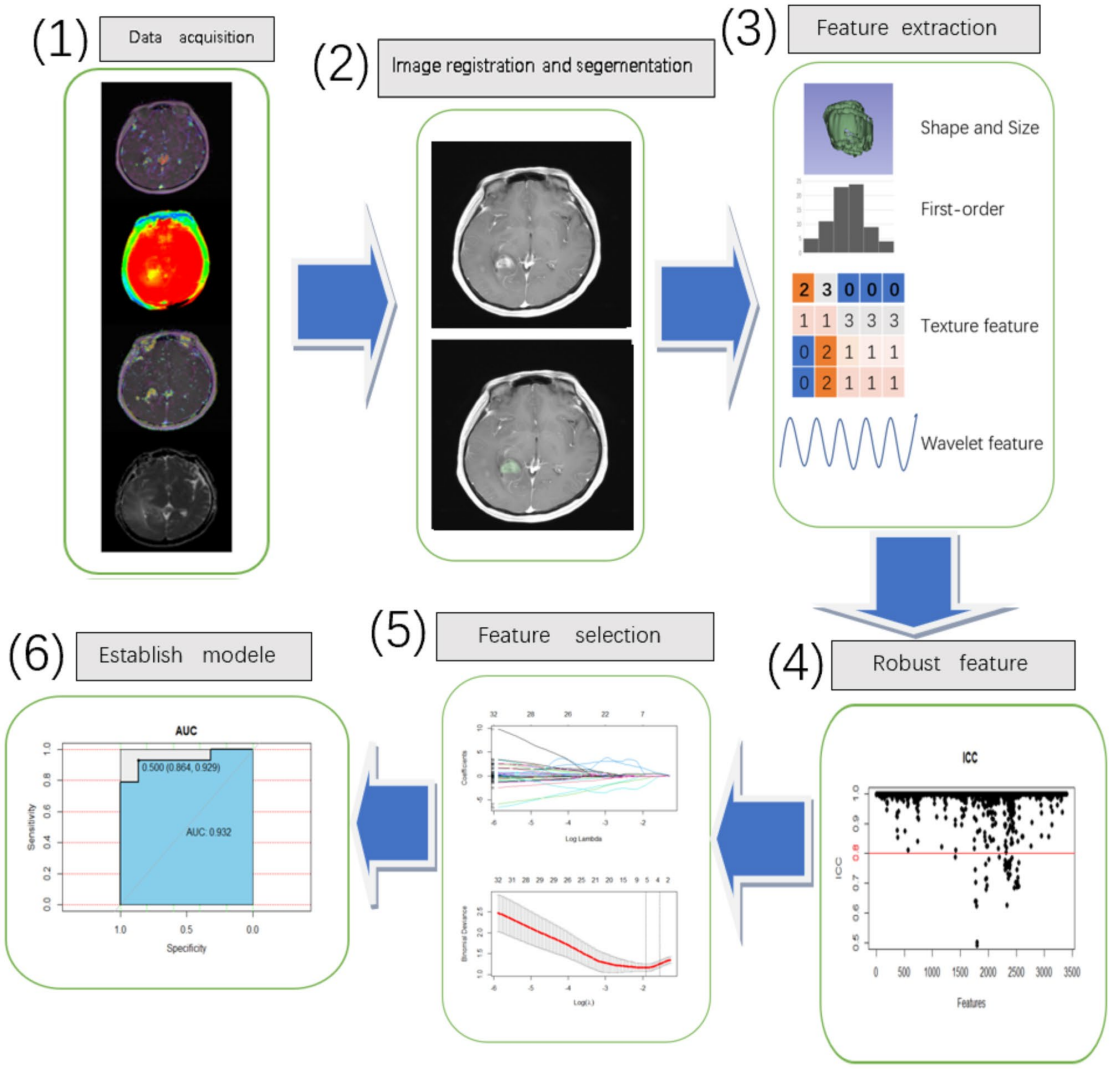
The flow chart of this study. (**1**): ADC, Ktrans, Ve and APTW images were co-registered with corresponding CE-T1WI images by 3D-Sliser. (**2**): Manually delineated the ROI of solid part of tumors. (**3**): Extracted the radiomics features. (**4**): Retained the features with ICC > 0.8. (**5**): Used the LASSO to select features. (**6**): Constructed single and combined sequence radiomics models and compared the AUC, sensitivity, specificity and accuracy of different models

Radial basis function kernel support vector machine (RBF-SVM) was used as classifier. Support vector machine (SVM) is a powerful binary classifier widely used in disease identification, gene typing and prognosis prediction [15–17]. Based on the features selected from the training set, we adjusted parameters (gamma and cost) in RBF-SVM and used SVM to establish the model. For predicting IDH-1 mutation state and Ki-67 expression, four single sequence models (ADC model, Ktrans model, Ve model, APTW model) and one combined model (ADC + Ktrans + Ve + APTW model) were established by SVM. The prediction models produced by the training set were evaluated using the receiver operator characteristic (ROC) curve and the area under the curve (AUC), sensitivity, specificity, accuracy, positive and negative prediction rates, and further evaluated with the validation set. Figure 3 was the flow chart describing this study.

### Statistical analysis

Statistical analyses were performed using SPSS 22.0 (IBM SPSS Statistics, version 22, Chicago, IL, USA) and R STUDIO (version 4.1.1, R package includes “e1071”, " pROC “, “glmnet”, etc.). The measurement data were expressed as means ± SD. Independent sample t-test was used for comparison between groups, and χ^2^ test was used for classification data when evaluating the differences of gender, age, Ki-67 expression and IDH-1 mutation status in training and validation sets. The Delong test was used to compare the differences in AUC between different models in the validation set. If *P* < 0.05, the differences were statistically significant.

## Results

### Demographics and clinical characteristics of the patients

Table 1 showed the demographics and clinical characteristics of the patients. There were no significant differences in clinical characteristics between the training and validation sets in the Ki-67 and IDH-1 groups (*P* > 0.05).

**Table 1:** Demographics and clinical characteristics of the patients

Characters	KI-67 group		*P*	IDH-1 group			*P*
	Training set (*N* = 216)	Validation set (*N* = 93)		Training set (*N* = 216)		Validation set (*N* = 93)	
Gender			>0.05				>0.05
Male	117	57		108		48	
Female	99	36		108		45	
Age(years)	42.36 ± 10.90	44.36 ± 12.90	>0.05	41.36 ± 10.42		40.36 ± 11.93	>0.05
Ki-67(-)	43.06%(93/216)	41.94 (36/93)	>0.05	-		-	
Ki-67(+)	56.94%(123/216)	58.06%(54/93)	>0.05	-		-	
IDH-1(+)	-	-		40.28%(87/216)		38.71%(36/93)	>0.05
IDH-1(-)	-	-		59.72%(129/216)		61.29%(57/93)	>0.05

Note Ki-67(-): low expression of Ki-67, Ki-67(+): high expression of Ki-67

### Robustness and selection of radiomic features

The features with ICC > 0.8 were retained, ADC, APTW, Ktrans and Ve retained 851, 850, 804 and 851 features, respectively. The independent sample t-test and Mann-Whitney U test were performed. In Ki-67 group, ADC, APTW, Ktrans and Ve images showed 352, 366, 412 and 442 tumor features, respectively (all *P* < 0.05). Combined sequence images showed 1511 tumor features (*P* < 0.05). Then, LASSO was used to select 6, 7, 7, 8, and 6 radiomics features for ADC, APTW, Ktrans, Ve and combined images, respectively. In IDH-1 group, ADC images showed 367 tumor features, APTW images showed 323 tumor features, Ktrans images showed 478 tumor features, and Ve images showed 321 tumor features (all *P* < 0.05). Combined sequence images showed 1516 tumor features (*P* < 0.05). Then LASSO was used to select 2, 5, 5, 6 and 4 radiomics features for ADC, APTW, Ktrans and Ve images and combined sequence images respectively. The four single sequence characteristics of ADC, APTW, Ktrans and Ve in the two groups were shown in the Table 2(A, B, C, D). Table 3 listed selected radiomic features from the combined sequence images in both groups.

**Table 2A:** The radiomic features of ADC in Ki-67 group and IDH-1 group

Group	MRI sequences	Feature number	Individual features
Ki-67	ADC	6	ADC - waveletLHL-glcm-Correlation (*P* = 0.0008)
			ADC -waveletLHH-glcm-Id(*P* = 0.006)
			ADC -waveletLHH-glcm-Idm (*P* = 0.0057)
			ADC -waveletLHH-ngtdm-Contrast (*P* = 0.014)
			ADC -waveletHLH-glszm-LowGrayLevelZoneEmphasis (*P* = 0.00189)
			ADC -waveletHHL-firstorder-Skewness(*P* = 0.005)
IDH-1	ADC	2	ADC -waveletLHH-glcm-DifferenceEntropy (*P* = 0.02)
			ADC -waveletHHL-glszm-ZoneVariance (*P* = 0.01)

**Table 2B:** The radiomic features of APTW in Ki-67 group and IDH-1 group

Group	MRI sequences	Feature number	Individual features
Ki-67	APTW	7	APTW-original-ngtdm-Coarseness (*P* = 0.005)
			APTW-waveletLLH-glcm-MaximumProbability(*P* = 0.009)
			APTW-waveletLHH-glszm-LowGrayLevelZoneEmphasis (*P* = 0.003)
			APTW-waveletHLL-firstorder-Median (*P* = 0.01)
			APTW-waveletHLH-glszm-ZoneEntropy (*P* = 0.005)
			APTW-waveletHHH-glcm-DifferenceAverage(*P* = 0.018)
			APTW-waveletHHH-glcm-InverseVariance(*P* = 0.018)
IDH-1	APTW	5	APTW-waveletHLL-glrlm-ShortRunEmphasis(*P* = 0.009)
			APTW- waveletLHL-glszm-ZoneVariance (*P* = 0.01)
			APTW- waveletHLH-glcm-Imc1 (*P* = 0.03)
			APTW- waveletHHH-firstorder-Skewness (*P* = 0.03)
			APTW- waveletHLH-ngtdm-Complexity (*P* = 0.02)

**Table 2C:** The radiomic features of ktrans in Ki-67 group and IDH-1 group

Group	MRI sequences	Feature number	Individual features
Ki-67	Ktrans	7	ktrans-waveletLHL-firstorder-Skewness (*P* = 0.04)
			ktrans-original-gldm-DependenceVariance(*P* = 0.002)
			ktrans-waveletLHH-firstorder-Mean(*P* = 0.003)
			ktrans-original-glcm-JointAverage (*P* = 0.002)
			ktrans-waveletLHH-glcm-Imc1(*P* = 0.005)
			ktrans-original-shape-Maximum3Ddiameter(*P* = 0.013)
			ktrans-waveletLHL-firstorder-Mean(*P* = 0.005)
IDH-1	Ktrans	5	ktrans-waveletLLH-glcm-MaximumProbability (*P* = 0.001)
			ktrans-original-firstorder-RootMeanSquared (*P* = 0.002)
			ktrans-waveletHLL-glszm-ZoneVariance (*P* = 0.03)
			ktrans-waveletLLH-glszm-LargeAreaEmphasis (*P* = 0.03)
			ktrans-original-gldm-DependenceEntropy (*P* = 0.002)

**Table 2D:** The radiomic features of Ve in Ki-67 group and IDH-1 group

Group	MRI sequences	Feature number	Individual features
Ki-67	Ve	8	Ve-original-shape-Maximum3DDiameter (*P* = 0.013)
			Ve-waveletLHL-glcm-Correlation(*P* = 0.0013)
			Ve-waveletHLL-firstorder-Maximum (*P* = 0.0003)
			Ve-original-glcm-Imc2 (*P* = 0.01)
			Ve-waveletHLL-firstorder-Range (*P*<0.001)
			Ve-waveletHLH-glcm-ClusterShade(*P* = 0.02)
			Ve-waveletHHH-glszm-ZoneEntropy(*P* = 0.007)
			Ve-waveletLLL-firstorder-Maximum(*P*<0.001)
IDH-1	Ve	6	original-gldm-DependenceVariance*P* = 0.01)
			waveletLHL-firstorder-Mean(*P* = 0.01)
			waveletHLH-gldm-DependenceVariance(*P* = 0.006)
			waveletLHH-ngtdm-Contrast (*P* = 0.04)
			waveletHLH-gldm-DependenceNonUniformityNormalized (*P* = 0.008)
			waveletHHL-glcm-ClusterShade(*P* = 0.02)

**Table 3:** The radiomic features of combined sequence images in Ki-67 group and IDH-1 group

Group	MRI sequences	Feature number	Individual features
Ki-67	Ktrans + Ve + APTW + ADC	6	APTW- waveletHLH-glszm-ZoneEntropy(*P* = 0.005)
			ktrans-original-gldm-DependenceVariance(*P* = 0.002)
			ktrans-waveletLHL-firstorder-Mean(*P* = 0.005)
			ktrans-waveletLHH-glcm-Imc1(*P* = 0.005)
			Ve-waveletHLL-firstorder-Range(*P*<0.001)
			Ve-waveletLLL-firstorder-Maximum(*P*<0.001)
IDH-1	Ktrans + Ve + APTW + ADC	4	APTW-waveletHLL-glrlm-ShortRunEmphasis(*P* = 0.009)
			ADC-waveletHHL-glszm-ZoneVariance(*P* = 0.010)
			ktrans-original-firstorder-RootMeanSquared(*P* = 0.002)
			Ve-waveletHLH-gldm-DependenceVariance(*P* = 0.006)

### Performance of radiomics models

**Fig. 4:**
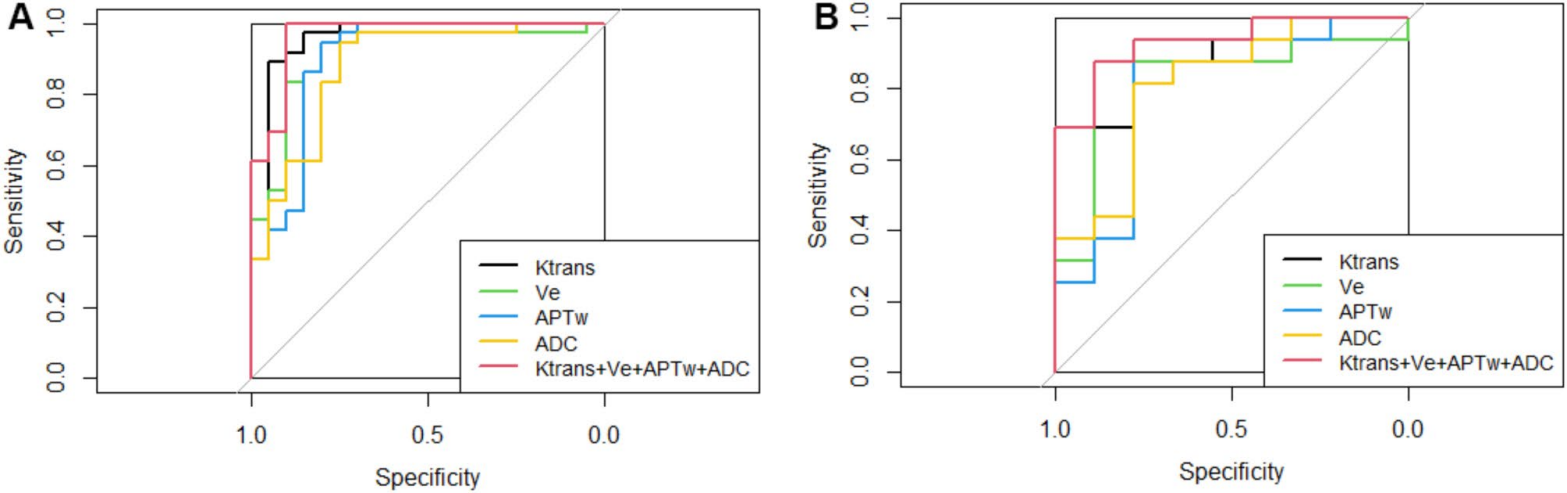
**A**: The ROC curve of Ki-67 training set model. **B**: The ROC curve of Ki-67 validation set model

**Fig. 5:**
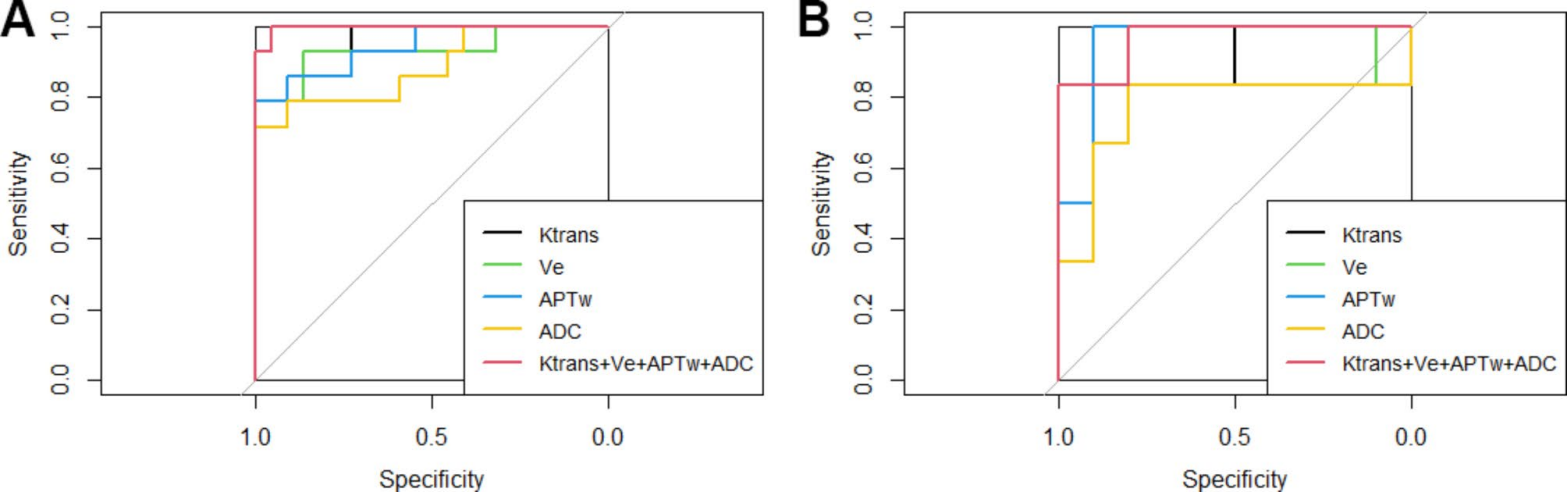
**A:** The ROC curve of IDH-1 training set model. **B**: The ROC curve of IDH-1 validation set model

For the Ki-67 group, the AUC of ADC, APTW, Ktrans, Ve and the combined model were 0.879, 0.900, 0.954, 0.910 and 0.965 in the training set, and 0.812, 0.812, 0.896, 0.833 and 0.931 in the validation set, respectively. The accuracy, sensitivity, and specificity of radiomics model were shown in Table 4. The ROC curve was shown in Fig. 4(A, B). For the IDH-1 group, the AUC of ADC, APTW, Ktrans, Ve and combined model were 0.899, 0.942, 0.964, 0.932 and 0.997 in the training set, and 0.783, 0.950, 0.883, 0.850 and 0.967 in the validation set, respectively. The accuracy, sensitivity, and specificity of the radiomics model were shown in Table 5. The ROC curve was shown in Fig. 5(A, B). In the Ki-67 group, the AUC of the combined model in validation set was compared to the AUC of ADC, APTW, Ktrans and Ve single-sequence model, the differences were statistically significant (all *P* < 0.05). In IDH-1 group, the AUC of the combined model in validation set was compared to the AUC of ADC, APTW, Ktrans and Ve single-sequence model, with *P* values of 0.032, 0.51, 0.043 and 0.037, respectively. Although the difference between the AUC of the combined model and the APTW model was not statistically significant in the validation set, the AUC, sensitivity and accuracy of the combined model were higher than those of the APTW model alone. In conclusion, both Ki-67 group, and IDH-1 group showed the best efficiency of the combined model.

**Table 4:** The effectiveness of each model in the Ki-67 group

Model	Group	AUC(95%CI)	SEN	SPE	ACC	PPV	NPV
Ktrans	Training set	0.954(0.887-1.000)	0.972	0.750	0.893	0.875	0.938
	Validation set	0.896(0.774-1.000)	0.750	0.778	0.760	0.857	0.636
Ve	Training set	0.910(0.824–0.995)	0.944	0.750	0.875	0.871	0.882
	Validation set	0.833(0.648-1.000)	0.875	0.889	0.880	0.933	0.800
APTW	Training set	0.900(0.801–0.999)	1.000	0.700	0.893	0.857	1.000
	Validation set	0.812(0.605-1.000)	0.938	0.667	0.840	0.833	0.857
ADC	Training set	0.879(0.780–0.978)	0.944	0.700	0.857	0.850	0.875
	Validation set	0.812(0.627–0.998)	0.875	0.444	0.720	0.737	0.667
Ktrans + Ve + APTW + ADC	Training set	0.965(0.916-1.000)	1.000	0.900	0.964	0.947	1.000
	Validation set	0.931(0.833-1.000)	0.875	0.889	0.880	0.933	0.800

**Table 5:** The effectiveness of each model in the IDH-1group

Model	Group	AUC(95%CI)	SEN	SPE	ACC	PPV	NPV
Ktrans	Training set	0.964(0.913-1.000)	0.857	0.864	0.861	0.800	0.905
	Validation set	0.883(0.700-1.000)	0.833	0.800	0.813	0.714	0.889
Ve	Training set	0.932(0.832-1.000)	0.786	0.909	0.861	0.846	0.870
	Validation set	0.850(0.554-1.000)	0.833	0.900	0.875	0.833	0.900
APTW	Training set	0.942(0.864-1.000)	0.786	1.000	0.917	1.000	0.880
	Validation set	0.950(0.843-1.000)	1.000	0.800	0.875	0.750	1.000
ADC	Training set	0.893(0.758-1.000)	0.714	0.955	0.861	0.909	0.840
	Validation set	0.817(0.596-1.000)	1.000	0.400	0.625	0.500	1.000
Ktrans + Ve + APTW + ADC	Training set	0.997(0.988-1.000)	0.929	1.000	0.972	1.000	0.957
	Validation set	0.967(0.888-1.000)	1.000	0.900	0.938	0.857	1.000

## Discussion

This study showed that the SVM single sequence model and combined model based on DWI, DCE, and APTW had good diagnostic performance in predicting the expression of Ki-67 and IDH-1 mutation in glioma. The combined sequence model had the best diagnostic performance in both groups.

Sun et al. found that the tumor radiomics model based on T2WI could better predict the expression of Ki-67 in glioma, and the sensitivity, specificity and AUC were 0.818, 0.833 and 0.773, respectively [18]. The study of Yiming Li et al. demonstrated that radiomic features based on T2WI could efficiently and non-invasively predict Ki-67 expression and survival in lower-grade gliomas [19]. The study of GAO et al. on using CE-T1WI sequence combined with machine learning algorithms to distinguish the expression of Ki-67 in glioma achieved good results, and the sensitivity, specificity and AUC were 0.91, 0.80 and 0.85, respectively [20]. However, most of these radiomics studies were limited to conventional MRI. Conventional MRI sequences, such as T1WI and T2WI, mostly reflect the anatomical information of tumors, CE-T1WI sequence can partly reflect the blood supply of tumors. Still, the influence of the blood-brain barrier should also be considered. MR perfusion techniques, DCE, can reflect the density of microvessels and vascular permeability of tumor. In our study, radiomics model based on DCE had high value for predicting the expression of Ki-67 in glioma. ADC reflects the degree of limited diffusion of water molecules, to some extent, it reflects the density of tumor. In our study, compared with the ADC, the AUC of DCE model for predicting the expression of Ki-67 in glioma was higher, whether in the training set or the verification set. Studies have shown that the ability of DWI sequence to distinguish glioma heterogeneity was weaker than that of DCE sequence [21], which was consistent with our study. However, DCE needs to inject the contrast agent intravenously. APTW is a novel, noninvasive MRI sequence, and can measure the endogenous moving proteins and peptides, which indirectly reflects the changes in the internal environment. The study of Yuan Li et al. concluded that APTW imaging was a feasible approach for detecting Ki-67 expression of type I EC, and the Ki-67 positively moderately correlates with APTW [22]. In the study on rectal adenocarcinoma, APT was positively related to Ki-67 expression in rectal adenocarcinoma. APT imaging may serve as a noninvasive biomarker for assessing genetic prognostic factors of rectal adenocarcinoma [23]. However, the radiomics based on APTW study on Ki-67 expression in tumors have not been reported. Our study about radiomics based on APTW showed that APTW had high predictive value for the expression of Ki-67 in glioma, and the AUC in the training set and validation set were 0.900 and 0.812, respectively. Although each single sequence model had high predictive value for Ki-67 expression in glioma, the diagnostic performance of our combined sequence model was better than that of previous studies, which was consistent with our hypothesis.

IDH is an important basis for the pathological classification of gliomas and reflects the patients’ prognosis. In recent years, more and more radiomics studies have been conducted on IDH, and the radiomics studies on the combination of fMRI are a current research hotspot [24]. The radiomics model based on T1 enhancement, T2WI and ASL sequences achieved good results in predicting IDH mutation of glioma, and the sensitivity, specificity and AUC were 0.765, 0.776 and 0.823, respectively [25]. Studies have shown that the combination of T1 enhancement, T2-FLAIR, DWI and DSC sequences can better predict IDH mutation in low-grade glioma, and the sensitivity, specificity, and AUC values were 0.707, 0.800, and 0.795, respectively [26]. Wang et al. established an SVM model combining DCE and DWI sequences, which predicted the IDH-1 mutation of glioma wonderfully, and the sensitivity, specificity and AUC were 0.893, 0.976, and 0.939, respectively [27]. All these studies indicated that the combination of MR perfusion techniques and radiomics had significant advantages in reflecting the IDH mutation status of glioma. In our study, both Ktrans and Ve single-parameter models of DCE achieved good diagnostic performance, which was consistent with previous studies. However, it is still limited to reflecting tumor heterogeneity only by the degree of tumor vascular proliferation and cell density. The study of Guo et al. on using APTW sequence and four diffusion-weighted sequences to distinguish IDH mutation status, showed that APTW was a very valuable MR sequence to identify IDH mutation status [28]. Hou H et al. stated that 3D-APTW and 3D-pCASL imaging could be used to evaluate IDH mutation status, and the diagnostic performance could be improved by combining the two techniques [29]. However, the hotspot research of APTW was relatively subjective and could not fully exploit imaging information. Han et al. established an SVM model based on APTW sequence, which achieved a good effect in discriminating the IDH mutation status of glioma, and the accuracy and AUC were 0.892 and 0.952 [30]. Nevertheless, the available radiomics information obtained only based on a single sequence, which does not well reflect the heterogeneity of tumors. Radiomic studies on the combination of DWI, DCE and APTW sequences have not been reported at present. Therefore, combined with the hot spots of previous studies, we established the SVM model based on DWI, DCE and APTW sequences to predict IDH mutation status, and achieved the best prediction effect. At the same time, the diagnostic performance of the combined model was better than that of the single sequence model, which indicated that the three sequence combinations did have a complementary role in the single sequence diagnosis of IDH-1 mutation.

This study consisted of two independent parts (IDH-1 and Ki-67), and each part adopted stratified sampling, which greatly reduced the sampling error. In the study of glioma grading based on ADC map, Lee et al. showed that avoiding necrotic and cystic areas could better distinguish high-grade and low-grade gliomas [31]. The differentiation of glioma heterogeneity by DWI, DCE and APTW sequences is based on the cell density, the proliferation of blood vessels and the content of mobile proteins in solid areas of tumor, while the necrotic and cystic areas reflect less information. Therefore, we selected the solid area of the tumor as the ROI to minimize the interference of the necrotic and cystic areas. At the same time, this study also existed some limitations. Firstly, because the patient needed a complete image of all three fMRIs, the sample size was relatively small. Then, including other clinical characteristics (age, gender, etc.) is extremely easy to make the model fitting. Therefore, this study mainly compared the diagnostic performance of different image models, we will expand the sample size for the later research. Moreover, clinical factors will be included to try to build a clinical-imaging comprehensive model. Secondly, this study was a single-center study, and it would be more rigorous if collect data from other centers were to form a test group. Finally, this study selected only the tumour itself as the ROI. The study of Li et al. on the radiomics of glioma established the intratulotumor multi-region model and peritulotumor model, and the results showed that the radiomics model containing multiple regions was better than the single-region model for predicting the mutation status of IDH-1 [32], which opened up ideas for subsequent ROI delineation.

## Conclusion

SVM models based on DWI, DCE and APTW were highly significant for evaluating the expression of Ki-67 and IDH-1 mutation status in glioma. The combined model of three sequences was better than the single sequence model and could better reflect glioma heterogeneity.

## Data Availability

Sequence data that support the findings of this study have been provided within the manuscript.

## Abbreviations

WHO: World Health Organization
IDH: Isocitrate dehydrogenase
fMRI: Functional magnetic resonance imaging
DCE: Dynamic contrast-enhanced
DWI: Diffusion-weighted imaging
APTW: Aminoacyl proton transfer-weighted
FOV: Field of view
FA: Flip Angle
TSE: Turbo Spin Echo
RF: Radio frequency
ROI: Region of interest
ICC: Intra-group correlation coefficient
SVM: Support vector machine
ROC: Receiver operator characteristic
AUC: Area under the curve

## References

[1] Pierscianek D, Ahmadipour Y, Oppong, et al. Blood-based biomarkers in high Grade gliomas: a systematic review. Mol Neurobiol. 2019;56(9):6071–9. 10.1007/s12035-019-1509-2. 30719642 10.1007/s12035-019-1509-2

[2] Louis DN, Perry A, Wesseling P, et al. The 2021 WHO classification of tumors of the Central Nervous System: a summary. Neuro Oncol. 2021;23(8):1231–51. 10.1093/neuonc/noab106. 34185076 10.1093/neuonc/noab106 PMC8328013

[3] Berger TR, Wen PY, Lang-Orsini M, Chukwueke UN. World Health Organization 2021 Classification of Central Nervous System Tumors and implications for Therapy for adult-type gliomas: a review. JAMA Oncol. 2022;8(10):1493–501. 10.1001/jamaoncol.2022.2844. 36006639 10.1001/jamaoncol.2022.2844

[4] Chen WJ, He DS, Tang RX, Ren FH, Chen G. Ki-67 is a valuable prognostic factor in gliomas: evidence from a systematic review and meta-analysis. Asian Pac J Cancer Prev. 2015;16(2):411–20. 10.7314/apjcp.2015.16.2.411. 25684464 10.7314/apjcp.2015.16.2.411

[5] Hu Y, Chen Y, Wang J, Kang JJ, Shen DD, Jia ZZ. Non-invasive estimation of Glioma IDH1 mutation and VEGF expression by Histogram Analysis of Dynamic contrast-enhanced MRI. Front Oncol. 2020;10:593102. 10.3389/fonc.2020.593102. 33425744 10.3389/fonc.2020.593102 PMC7793903

[6] Choi HS, Kim AH, Ahn SS, Shin NY, Kim J, Lee SK. Glioma grading capability: comparisons among parameters from dynamic contrast-enhanced MRI and ADC value on DWI. Korean J Radiol. 2013;14(3):487–92. 10.3348/kjr.2013.14.3.487. 23690718 10.3348/kjr.2013.14.3.487 PMC3655305

[7] Zhou J, Payen JF, Wilson DA, Traystman RJ, van Zijl PC. Using the amide proton signals of intracellular proteins and peptides to detect pH effects in MRI. Nat Med. 2003;9(8):1085–90. 10.1038/nm907. 12872167 10.1038/nm907

[8] Xu Z, Ke C, Liu J, et al. Diagnostic performance between MR Amide Proton transfer (APT) and difusion kurtosis imaging (DKI) in glioma grading and IDH mutation status prediction at 3T. Eur J Radiol. 2021;134:109466. 10.1016/j.ejrad.2020.109466. 33307459 10.1016/j.ejrad.2020.109466

[9] Lambin P, Leijenaar RTH, Deist TM, et al. Radiomics: the bridge between medical imaging and personalized medicine. Nat Rev Clin Oncol. 2017;14(12):749–62. 10.1038/nrclinonc.2017.141. 28975929 10.1038/nrclinonc.2017.141

[10] Huang WY, Wen LH, Wu G, et al. Comparison of Radiomics analyses based on different magnetic resonance imaging sequences in grading and molecular genomic typing of Glioma. J Comput Assist Tomogr. 2012;45(1):110–20. 10.1097/RCT.0000000000001114. 10.1097/RCT.0000000000001114 33475317

[11] Qian Z, Li Y, Wang Y, et al. Differentiation of glioblastoma from solitary brain metastases using radiomic machine-learning classifiers. Cancer Lett. 2019;451:128–35. 10.1016/j.canlet.2019.02.054. 30878526 10.1016/j.canlet.2019.02.054

[12] Han W, Qin L, Bay C, et al. Deep transfer learning and Radiomics Feature Prediction of Survival of patients with high-Grade Gliomas. AJNR Am J Neuroradiol. 2020;41(1):40–8. 10.3174/ajnr.A6365. 31857325 10.3174/ajnr.A6365 PMC6975328

[13] Cai J, Zhang C, Zhang W, et al. ATRX, IDH1-R132H and Ki-67 immunohistochemistry as a classification scheme for astrocytic tumors. Oncoscience. 2016;3:258–65. 10.18632/oncoscience.317. 27713914 10.18632/oncoscience.317 PMC5043074

[14] Kang J, Choi YJ, Kim IK, et al. LASSO-Based machine learning algorithm for prediction of Lymph Node Metastasis in T1 colorectal Cancer. Cancer Res Treat. 2021;53(3):773–83. 10.4143/crt.2020.974. 33421980 10.4143/crt.2020.974 PMC8291173

[15] Yang Y, Yan LF, Zhang X, et al. Optimizing texture Retrieving Model for Multimodal MR Image-based support Vector Machine for classifying glioma. J Magn Reson Imaging. 2019;49(5):1263–74. 10.1002/jmri.26524. 30623514 10.1002/jmri.26524

[16] Chen H, Li C, Zheng L, Lu W, Li Y, Wei Q. A machine learning-based survival prediction model of high grade glioma by integration of clinical and dose-volume histogram parameters. Cancer Med. 2021;10(8):2774–86. 10.1002/cam4.3838. 33760360 10.1002/cam4.3838 PMC8026951

[17] Zhang X, Tian Q, Wang L, et al. Radiomics Strategy for Molecular Subtype Stratification of Lower-Grade Glioma: detecting IDH and TP53 mutations based on Multimodal MRI. J Magn Reson Imaging. 2018;48(4):916–26. 10.1002/jmri.25960. 29394005 10.1002/jmri.25960

[18] Sun X, Pang P, Lou L, Feng Q, Ding Z, Zhou J. Radiomic prediction models for the level of Ki-67 and p53 in glioma. J Int Med Res. 2020;48(5):300060520914466. 10.1177/0300060520914466. 32431205 10.1177/0300060520914466 PMC7241212

[19] Li Y, Qian Z, Xu K, et al. Radiomic features predict Ki-67 expression level and survival in lower grade gliomas. J Neurooncol. 2017;135(2):317–24. 10.1007/s11060-017-2576-8. 28900812 10.1007/s11060-017-2576-8

[20] Gao M, Huang S, Pan X, Liao X, Yang R, Liu J. Machine learning-based Radiomics Predicting Tumor grades and expression of multiple pathologic biomarkers in Gliomas. Front Oncol. 2020;10:1676. 10.3389/fonc.2020.01676. 33014836 10.3389/fonc.2020.01676 PMC7516282

[21] Arevalo-Perez J, Peck KK, Young RJ, Holodny AI, Karimi S, Lyo JK. Dynamic contrast-enhanced perfusion MRI and diffusion-weighted imaging in Grading of Gliomas. J Neuroimaging. 2015;25(5):792–8. 10.1111/jon.12239. 25867683 10.1111/jon.12239 PMC5525149

[22] Li Y, Lin CY, Qi YF, et al. Three-dimensional turbo-spin-echo amide proton transfer-weighted and intravoxel incoherent motion MR imaging for type I endometrial carcinoma: correlation with Ki-67 proliferation status. Magn Reson Imaging. 2021;78:18–24. 10.1016/j.mri.2021.02.006. 33556484 10.1016/j.mri.2021.02.006

[23] Li L, Chen W, Yan Z, et al. Comparative Analysis of Amide Proton transfer MRI and diffusion-weighted imaging in assessing p53 and Ki-67 expression of rectal adenocarcinoma. J Magn Reson Imaging. 2020;52(5):1487–96. 10.1002/jmri.27212. 32524685 10.1002/jmri.27212

[24] Tan Y, Mu W, Wang XC, Yang GQ, Gillies RJ, Zhang H. Whole-tumor radiomics analysis of DKI and DTI may improve the prediction of genotypes for astrocytomas: a preliminary study. Eur J Radiol. 2020;124:108785. 10.1016/j.ejrad.2019.108785. 32004731 10.1016/j.ejrad.2019.108785

[25] Peng H, Huo J, Li B, et al. Predicting Isocitrate dehydrogenase (IDH) mutation status in Gliomas using Multiparameter MRI Radiomics features. J Magn Reson Imaging. 2021;53(5):1399–407. 10.1002/jmri.27434. 33179832 10.1002/jmri.27434

[26] Kim M, Jung SY, Park JE, et al. Diffusion- and perfusion-weighted MRI radiomics model may predict isocitrate dehydrogenase (IDH) mutation and tumor aggressiveness in diffuse lower grade glioma. Eur Radiol. 2020;30(4):2142–51. 10.1007/s00330-019-06548-3. 31828414 10.1007/s00330-019-06548-3

[27] Wang J, Hu Y, Zhou X, et al. A radiomics model based on DCE-MRI and DWI may improve the prediction of estimating IDH1 mutation and angiogenesis in gliomas. Eur J Radiol. 2022;147:110141. 10.1016/j.ejrad.2021.110141. 34995947 10.1016/j.ejrad.2021.110141

[28] Guo H, Liu J, Hu J, et al. Diagnostic performance of gliomas grading and IDH status decoding a comparison between 3D amide proton transfer APT and four diffusion-weighted MRI models. J Magn Reson Imaging. 2022;56(6):1834–44. 10.1002/jmri.28211. 35488516 10.1002/jmri.28211 PMC9790544

[29] Hou H, Chen W, Diao Y, et al. 3D Amide Proton transfer-weighted imaging for Grading Glioma and correlating IDH Mutation Status: added value to 3D pseudocontinuous arterial spin labelling perfusion. Mol Imaging Biol. 2023;25(2):343–52. 10.1007/s11307-022-01762-w. 35962302 10.1007/s11307-022-01762-w

[30] Han Y, Wang W, Yang Y, et al. Amide Proton Transfer Imaging in Predicting Isocitrate dehydrogenase 1 mutation status of Grade II/III Gliomas based on support Vector Machine. Front Neurosci. 2020;14:144. 10.3389/fnins.2020.00144. 32153362 10.3389/fnins.2020.00144 PMC7047712

[31] Lee J, Choi SH, Kim JH, Sohn CH, Lee S, Jeong J. Glioma grading using apparent diffusion coefficient map: application of histogram analysis based on automatic segmentation. NMR Biomed. 2014;27(9):1046–52. 10.1002/nbm.3153. 25042540 10.1002/nbm.3153

[32] Li ZC, Bai H, Sun Q, et al. Multiregional radiomics profiling from multiparametric MRI: identifying an imaging predictor of IDH1 mutation status in glioblastoma. Cancer Med. 2018;7(12):5999–6009. 10.1002/cam4.1863. 30426720 10.1002/cam4.1863 PMC6308047

